# Surgical Treatment of Pubic Symphysis Instability Using Semitendinosus Tendon Grafts: A Two-Case Report with Surgical Technique Description

**DOI:** 10.1055/s-0046-1820487

**Published:** 2026-06-16

**Authors:** Henrique Antônio Berwanger de Amorim Cabrita, Tatiana Ciocler Trahtenberg Guttmann, Adriano Rodrigues da Silva

**Affiliations:** 1 Institute of Orthopedics and Traumatology, Hospital das Clínicas, Faculdade de Medicina, Universidade de São Paulo, São Paulo, SP, Brazil; 2 Department of Orthopedics and Traumatology, Hospital Israelita Albert Einstein, São Paulo, SP, Brazil

**Keywords:** hamstring tendons, joint instability, osteitis, pubic bone, symphysis pubis, instabilidade articular, osso púbico, osteíte, sínfise púbica, tendões dos músculos isquiotibiais

## Abstract

The pubic symphysis is a key joint for the stability of the pelvic ring. Pubic symphysis instability may lead to chronic pain and significant functional limitation, particularly in athletes and young subjects. Although conservative management is the first-line approach, refractory cases may require surgical intervention. The present study aimed to describe a surgical technique used in two soccer players who underwent pubic symphysis reconstruction through a Pfannenstiel approach with an autologous semitendinosus tendon graft passed through bone tunnels in the pubic bones in a figure- eight configuration and fixed with non-absorbable sutures. This technique's goal is to preserve the physiological mobility of the pubic symphysis. At 6 months, both patients showed an improvement of more than 30 points in the modified Harris Hip Score. Moreover, they achieved satisfactory functional recovery, returning to sports at 6 and 8 months, respectively. Radiographic evaluation demonstrated improved pelvic stability over a 5-year follow-up period. The technique proved to be safe, with low morbidity, and good clinical outcomes, allowing pelvic mobility preservation and return to sports. Despite the small number of cases, this surgical approach appears to be reproducible, with biomechanical advantages over pubic symphysis arthrodesis and a low complication rate.

## Resumo

A sínfise púbica é uma articulação fundamental para a estabilidade do anel pélvico. A instabilidade desta articulação pode causar dor crônica e limitação funcional significativa, especialmente em atletas e indivíduos jovens. Embora o tratamento conservador seja a primeira linha de manejo, casos refratários podem exigir abordagem cirúrgica. Este estudo propõe apresentar uma descrição de técnica cirúrgica utilizada em dois pacientes atletas de futebol que foram submetidos à reconstrução da sínfise púbica com via de acesso de Pffanestiel, passagem de enxerto autólogo do 'tendão semitendinoso através de túneis ósseos nos ossos do púbis, configurados “em oito” e fixados com fios de sutura inabsorvíveis. A técnica visou preservar a mobilidade fisiológica da sínfise púbica. Após 6 meses, houve melhora de mais de 30 pontos do escore de Harris modificado para o quadril e retorno funcional satisfatório às atividades físicas com retorno ao esporte após 6 e 8 meses, respectivamente. Houve melhora da estabilidade pélvica radiológica durante o seguimento de 5 anos. A técnica demonstrou ser segura, com baixa morbidade e bons resultados clínicos, permitindo a preservação da mobilidade pélvica e o retorno ao esporte. Apesar do baixo número de casos, esta alternativa cirúrgica é reprodutível, com vantagens biomecânicas em comparação com a artrodese da sínfise púbica e baixo índice de complicações.

## Introduction

The pubic symphysis, a joint that connects the pubic bones, is essential for the stability of the pelvic ring and for the transmission of forces between the trunk and the lower limbs. 1 Its dysfunction may lead to pain, functional limitation, and impaired athletic performance.

The origin of pubic symphysis instability may be traumatic, gestational, mechanical overload-related, or muscular imbalance-related. In athletes, particularly those involved in sports that require sudden changes of direction or long-distance running, repetitive microtrauma may lead to chronic instability of the pubic symphysis. 2 3

From a biomechanical perspective, the pelvis links ascending and descending forces due to the action of antagonistic muscle groups such as the adductors, abdominal muscles, and hamstrings. Imbalance among these vectors may generate shear and rotational forces on the pubic symphysis, leading to microinstability, inflammation, and, in chronic cases, degeneration of the interpubic disc. 2

The diagnosis of pubic symphysis instability is a challenge. Symptoms are often nonspecific and may mimic other causes of hip-related pain, such as femoroacetabular impingement and osteitis pubis. Clinical evaluation, combined with imaging studies—particularly dynamic flamingo-view radiographs and magnetic resonance imaging (MRI)—is essential for accurate diagnosis. 1 4 5 6

Conservative treatment is the first-line approach and includes physical therapy, analgesia, and rest. Despite these measures, some cases—particularly those with radiographic instability greater than 5 mm—remain symptomatic and require surgical intervention. 7 Arthrodesis, although effective in eliminating instability, may compromise pelvic biomechanics and result in functional limitation. 4 In addition, complication rates of up to 25% have been reported in some case series, including sacroiliac joint degeneration due to abnormal pelvic biomechanics. 1 In this context, ligament reconstruction using autologous grafts has emerged as a promising alternative, with the potential to restore stability while preserving the physiological mobility of the joint.

The present study aims to describe a novel surgical technique for pubic symphysis reconstruction using autologous hamstring tendon grafts in two patients with clinically and radiologically confirmed pubic symphysis instability refractory to conservative treatment. The study was approved by the Research Ethics Committee (CAAE No. 90192625.4.0000.0071), and all participants provided written informed consent.

**Fig. 1:**
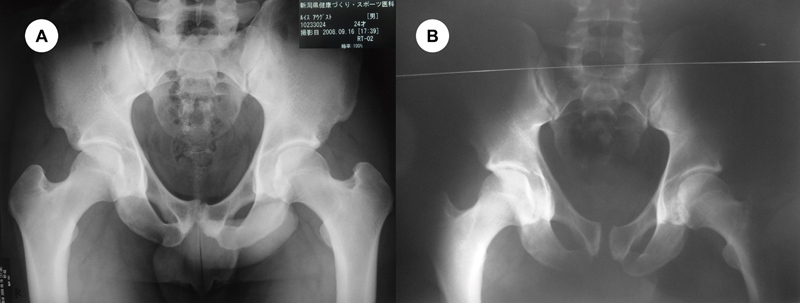
( **A,B** ) Anteroposterior and inlet radiographs of the pelvis demonstrating cortical irregularity and narrowing of the suprapubic joint space.

The first case involved a 26-year-old male high-performance soccer player with chronic pubic pain since 2007, associated with a limping gait and hip limitation. On hip examination, the patient reported pain during anterior and posterior impingement tests, as well as during the Patrick, Grava, and Valsalva tests. Imaging studies ( Fig. 1 ) demonstrated juxtacortical bony changes at the surface of the pubic symphysis, and MRI revealed capsular and peritendinous lesions. After failure of conservative treatment—including infiltrations and three prior surgical procedures (adductor tenotomy, inguinal hernia repair, and pubic symphysis resection)—the patient underwent pubic symphysis reconstruction using an autologous semitendinosus tendon graft. Dynamic flamingo-view radiographs showed a vertical displacement of the pubic rami of 25 mm (normal ≤ 5 mm), consistent with pubic instability, as well as a 20 mm diastasis of the pubic symphysis. 7 During the same surgical procedure, right hip arthroscopy was performed for labral repair and correction of a cam-type deformity. The modified Harris Hip Score improved from 45 preoperatively to 92 at 6 months and 90 at 5 years of follow-up. Return to professional sports occurred 6 months after surgery.

The second case involved a 22-year-old male professional soccer player with progressive pubic pain for 6 months. The patient had negative tests for intra-articular hip pain but presented pain localized to the pubic symphysis. Symptoms were refractory to 6 months of conservative treatment, with associated limitation in sports performance and a modified Harris Hip Score of 58. Dynamic flamingo-view radiographs demonstrated a vertical displacement of 18 mm between the right and left pubic rami under load. Pubic symphysis reconstruction was performed due to symptom persistence. The patient returned to professional soccer after 8 months of rehabilitation, with improvement in the modified Harris Hip Score to 90 at 6 months and 97 at 5 years of follow-up.

### Surgical technique

**Fig. 2:**
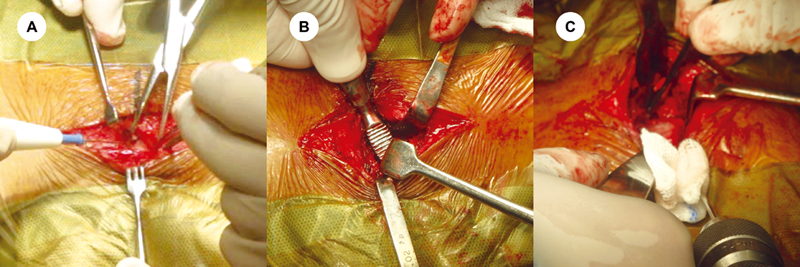
( **A** ) Pfannenstiel incision. ( **B** ) Layered dissection and muscle retraction. ( **C** ) Creation of bilateral bone tunnels in the pubic rami, approximately 1.5 cm lateral to the pubic symphysis, oriented from anteroinferior to posterosuperior.

All patients underwent the procedure under general anesthesia in the supine position. A transverse suprapubic Pfannenstiel incision, approximately 5 cm in length, was made about 2 cm proximal to the pubic tubercle ( Fig. 2A ). Layered dissection was carried out to expose the anterior fascia of the rectus abdominis muscle, which was incised longitudinally along the linea alba to allow lateral retraction of the rectus abdominis sheaths ( Fig. 2B ).

Careful manual dissection of the posterior aspect of the pubic symphysis was performed to avoid injury to the bladder and the retropubic venous plexus. A retractor was placed obliquely at the pubic tubercle to improve exposure of the distal portion of the rectus abdominis muscles.

The semitendinosus tendon from the non-dominant limb was harvested by a knee surgeon and used as the graft. Both ends of the graft were prepared with Krakow sutures to facilitate handling and fixation.

**Fig. 3:**
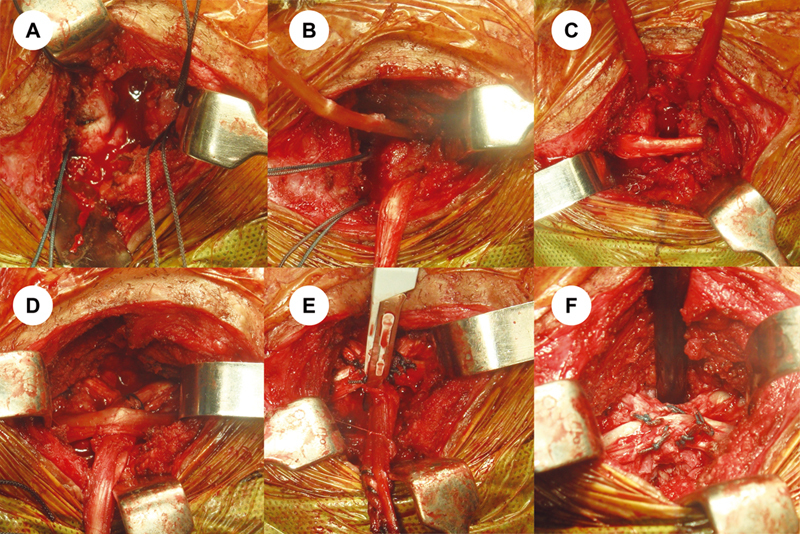
( **A** ) Positioning of the semitendinosus graft at the pubic symphysis. ( **B** ) Passage of the graft through the first bone tunnel in a craniocaudal direction. ( **C** ) U-shaped configuration of the graft through the tunnels. ( **D** ) Graft tensioning to reduce symphyseal diastasis and fixation in a figure-of-eight configuration. ( **E** ) Resection of excess graft. ( **F** ) Final appearance after graft fixation.

Bilateral bone tunnels were created in the pubic rami, approximately 1.5 cm lateral to the pubic symphysis, oriented from anteroinferior to posterosuperior ( Fig. 2C ). Using high-strength sutures, the graft was passed through the tunnels, forming a U-shaped configuration around the pubic symphysis: on the right side, in a craniocaudal direction; on the left side, in a caudocranial direction ( Figs. 3A–C ). The graft ends were tensioned posterior to the symphysis to reduce the diastasis. Fixation was then achieved by suturing the graft to itself under controlled tension using non-absorbable sutures, creating a figure-eight configuration ( Figs. 3D ). Excess graft was trimmed ( Fig. 3E ). At the end of the procedure, joint stability was assessed manually, confirming adequate graft tensioning and fixation ( Fig. 3F ).

Postoperatively, patients followed a structured rehabilitation protocol focused on pelvic mobility, functional recovery, and pain control using cryotherapy and neuromuscular electrical stimulation. Partial weight-bearing with a walker or two crutches was allowed for 2 months, followed by an additional month of protected weight-bearing with a single crutch on the more functionally affected side.

Progression of joint mobility was facilitated by continuous passive motion for four weeks, combined with guided kinesiotherapy.

Motor control activation was initiated after two months through core strengthening exercises, with gradual progression based on clinical tolerance and functional response.

Plyometric exercises on a trampoline were introduced at 4 months, followed by return to running at 5 months and progressive return to sports after 6 months.

**Fig. 4:**
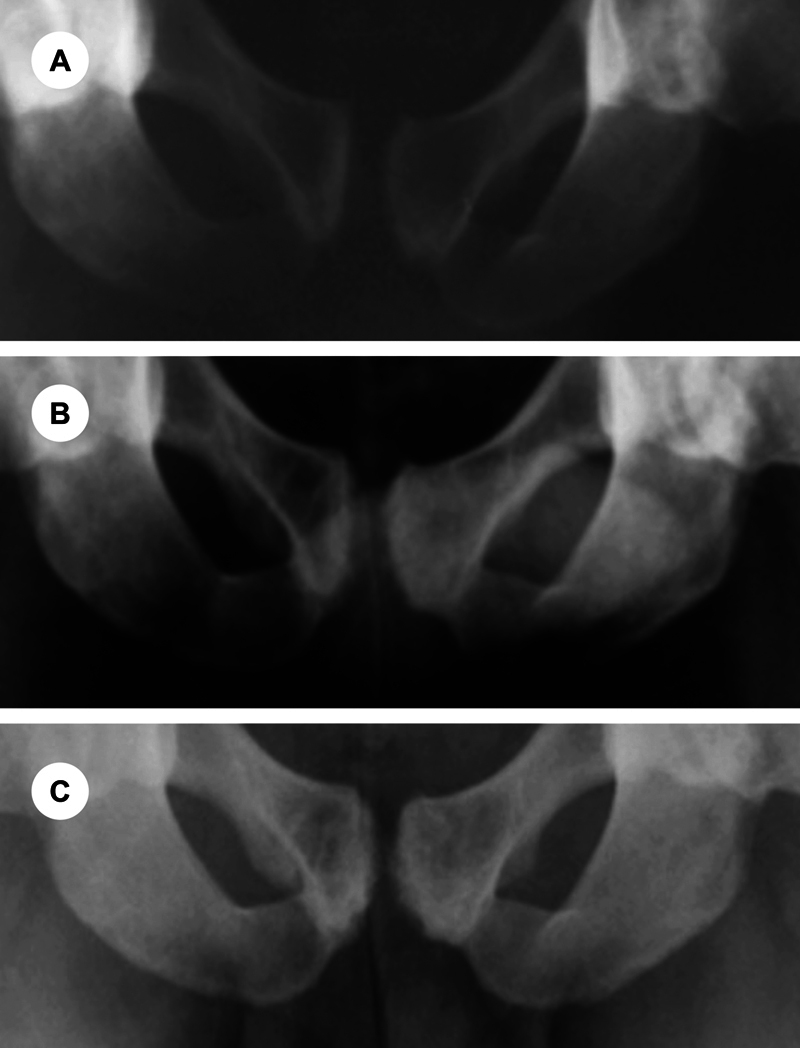
Serial radiographs of the pubic symphysis: ( **A** ) Preoperative; ( **B** ) postoperative at 6 months; ( **C** ) postoperative at 42 months.

Patients underwent serial evaluations to assess the clinical, functional, and structural progression of the pubic symphysis, as well as the effectiveness of the reconstruction using an autologous graft. Assessments included radiographic parameters, MRI, and clinical outcomes at preoperative baseline, 6 months, and 5 years postoperatively ( Fig. 4 ). Functional evaluation used the modified Harris Hip Score. For pain intensity assessment, the visual analog scale (VAS) was employed. Table 1 shows the progressive improvement observed in both cases.

**Table 1:** Pain and functional scores before surgery and at the 6-month and 5-year follow-ups

	VAS (pain)		Modified Harris Hip Score	
	Patient 1	Patient 2	Patient 1	Patient 2
Before surgery	10	8	45	58
6 months	2	0	92	90
5 years	0	0	90	97

**Abbreviation** : VAS, Visual Analog Scale.

As described, both patients returned to high-level sports activity without restrictions at 6 and 8 months postoperatively, respectively, with significant improvement in pain scores on VAS following the rehabilitation protocol. At 5-year follow-up, there was no recurrence of symptoms, sacroiliac pain, or procedure-related complications.

## Discussion

Injuries of the pubic symphysis represent a significant challenge in orthopedic practice, particularly when associated with pelvic instability or failure of previous conservative and surgical treatments.

In the first case described, the patient had previously undergone pubic symphysis resection, which led to worsening pelvic instability. This finding is consistent with the report by Moore et al., 8 who described cases of women with pelvic instability following pubic resection treated with arthrodesis of the pubic symphysis and both sacroiliac joints, achieving good functional outcomes at 2 years, despite residual low-back pain.

The second patient demonstrated pubic symphysis mobility greater than 5 mm on flamingo-view radiographs, consistent with pelvic instability according to the radiographic criteria described by Siegel et al. 7

Several surgical techniques have been described for the management of these injuries, including arthrodesis using plate fixation with or without bone grafting, as well as reconstructions using synthetic materials. 9 10 11 12 13

As this condition is rare, existing studies are limited to small case series, usually involving 1 to 8 patients, with reported success rates from 67% to 100%. 14

Arthrodesis using orthogonal plate fixation is the most described technique. In 2 case series of athletes, each including 8 patients and reviewed by Williams et al., 9 Choi et al., 15 and Vitanzo et al., 16 the outcomes at a mean follow-up of 52 months showed a complication rate of 25% and return to sport in 83% of cases.

Olucha-Puchol et al. 17 described a surgical technique involving arthrodesis with a single plate and metallic cerclage in two cases; however, these patients were non-athletic women over 50 years of age.

In our cases, particular concern was given to the potential consequences of arthrodesis in patients expected to maintain an active professional athletic career, especially regarding its impact on the biomechanics of the sacroiliac joints and lumbar spine. 9

Fixation failure is another important concern, being reported in 43% of 148 traumatic cases treated with dual-plate arthrodesis by Morris et al., 18 with 8% of patients requiring revision surgery.

Biomechanical studies using cadaveric pelvic ring models have demonstrated that physiological fixation with tape suture provides adequate stability without exceeding the normal mobility of approximately 2 mm of the pubic symphysis, even under cyclic vertical and horizontal loading conditions. 19

Castropil et al. 20 compared three techniques for reconstruction of sternoclavicular instability—an anatomically similar joint to the pubic symphysis—including intramedullary fixation, reinforcement with the subclavius tendon, and reconstruction with a semitendinosus tendon graft in a figure-eight configuration. Their results demonstrated superior biomechanical properties with the semitendinosus graft technique, supporting the application of this principle to pubic symphysis reconstruction.

The technique presented in this report is proposed as a biological alternative for treating chronic instability, as autologous hamstring tendon grafts exhibit viscoelastic properties that simulate ligament function, allowing them to resist shear and distraction forces while maintaining a degree of elasticity. 20

Similar to the technique described by Arner et al., 5 which involves laparoscopic fixation using anchors and tape suture in a crossed configuration, pubic symphysis reconstruction using hamstring grafts aims to restore joint stability while preserving physiological mobility, thereby mimicking ligament function and meeting the specific functional demands of the patient.

Preservation of pubic symphysis mobility is particularly relevant in active individuals and athletes, as the micromovements of this joint are essential for activities involving running, jumping, and directional changes. 12 Arthrodesis, in contrast, completely eliminates this mobility, altering movement patterns and increasing mechanical stress on adjacent structures, such as the sacroiliac joints and lumbar spine, which may compromise athletic performance. 4 13

A potential limitation of using an autologous semitendinosus tendon graft is the possible reduction in knee flexion strength. However, this tendon is widely used in ligament reconstruction procedures due to its availability, strength, and low complication rates. In both cases, postoperative isokinetic testing demonstrated normal flexor/extensor strength ratios, with no reported hamstring injuries during long-term follow-up. An alternative approach would be the use of allografts, allowing biological reconstruction without donor-site morbidity. 6

Functional outcomes, as assessed by the modified Harris Hip Score, demonstrated significant improvement, with increases of more than 30 points at 6 months in both cases.

Pain improvement, as measured by VAS at 6 months and 5 years of follow-up, further supports the positive impact of this technique on patient quality of life.

These findings suggest that this surgical approach may expand current treatment options for anterior pubic symphysis instability and support the use of a previously undescribed technique with the potential to restore both stability and physiological mobility, even at mid-term follow-up (5 years).

However, this study includes only two patients, reflecting the limited data available in the literature, which is largely restricted to small case series due to the rarity of the condition. Further studies with larger sample sizes—preferably prospective, controlled designs with long-term follow-up—are needed to validate these findings.

## Conclusion

Clinical improvement and return to physical activity were observed, with maintenance of radiographic pelvic stability over a 5-year follow-up period. Reconstruction using an autologous semitendinosus tendon graft proved to be a safe technique with low morbidity and favorable functional outcomes, as demonstrated by the modified Harris Hip Score in these two professional soccer players. However, studies with larger sample sizes are necessary to confirm and reproduce these findings.
